# Prostate Cancer Secretome and Membrane Proteome from *Pten* Conditional Knockout Mice Identify Potential Biomarkers for Disease Progression

**DOI:** 10.3390/ijms23169224

**Published:** 2022-08-17

**Authors:** Nilton J. Santos, Ana Carolina Lima Camargo, Hernandes F. Carvalho, Luis Antonio Justulin, Sérgio Luis Felisbino

**Affiliations:** 1 Laboratory of Extracellular Matrix Biology, Department of Structural and Functional Biology, Institute of Biosciences of Botucatu (IBB), São Paulo State University (UNESP), Botucatu 18618-689, SP, Brazil; 2 Laboratory of Extracellular Matrix and Gene Regulation, Department of Structural and Functional Biology, Institute of Biology (IB), University of Campinas (UNICAMP), Campinas 13083-862, SP, Brazil; 3 Laboratory of Human Genetics, Center for Molecular Biology and Genetic Engineering (CBMEG), University of Campinas (UNICAMP), Campinas 13083-875, SP, Brazil

**Keywords:** prognostic biomarkers, prostate cancer, transcriptomic-based secretome

## Abstract

Prostate cancer (PCa) is the second most common cause of mortality among men. Tumor secretome is a promising strategy for understanding the biology of tumor cells and providing markers for disease progression and patient outcomes. Here, transcriptomic-based secretome analysis was performed on the PCa tumor transcriptome of Genetically Engineered Mouse Model (GEMM) *Pb-Cre4/Pten^f/f^* mice to identify potentially secreted and membrane proteins—PSPs and PMPs. We combined a selection of transcripts from the GSE 94574 dataset and a list of protein-coding genes of the secretome and membrane proteome datasets using the Human Protein Atlas Secretome. Notably, nine deregulated PMPs and PSPs were identified in PCa (*DMPK*, *PLN*, *KCNQ5*, *KCNQ4*, *MYOC*, *WIF1*, *BMP7*, *F3*, and *MUC1*). We verified the gene expression patterns of Differentially Expressed Genes (DEGs) in normal and tumoral human samples using the GEPIA tool. *DMPK*, *KCNQ4*, and *WIF1* targets were downregulated in PCa samples and in the GSE dataset. A significant association between shorter survival and *KCNQ4*, *PLN*, *WIF1*, and *F3* expression was detected in the MSKCC dataset. We further identified six validated miRNAs (mmu-miR-6962-3p, mmu-miR- 6989-3p, mmu-miR-6998-3p, mmu-miR-5627-5p, mmu-miR-15a-3p, and mmu-miR-6922-3p) interactions that target *MYOC*, *KCNQ5*, *MUC1*, and *F3*. We have characterized the PCa secretome and membrane proteome and have spotted new dysregulated target candidates in PCa.

## 1. Introduction

Prostate cancer (PCa) is the most frequent cancer and has the second-highest morbidity and mortality rate among men, with 1,276,106 (7.1%) new cases globally and 358,989 (3.8%) deaths by cancer [1,2]. In the United States, the estimated number of new cases of PCa diagnosed in 2021 was 248,530, with 34,130 deaths. PCa in the United States accounts for 26% of all new cancer cases [3]. Current statistics show that one in seven men will be diagnosed with prostate cancer during their life and that one in 39 men will die of the disease [4].

The introduction of novel androgen receptor (AR) antagonists for clinical treatment has improved outcomes; however, most metastatic castration-resistant prostate cancer (mCRPC) patients ultimately develop resistance to these therapies. Patients with localized and advanced prostate tumors are sensitive to androgen deprivation therapy (ADT) and are highly curable; patients with metastatic prostate cancer acquire resistance to ADT and succumb to this disease [5]. While a large number of prostate cancer cases are diagnosed at a localized stage and are curable, metastatic prostate cancer remains fatal. In the last decade, large-scale omics analysis has revealed well-established and new master regulators and pathways involved in the metastatic and lethal behavior of PCa [6]. mCRPC is incurable, with a median survival rate f two years from diagnosis, and available treatments extend life for few months [7].

mCRPC commonly exhibits genetic alterations involving the AR, cell cycle and cell survival pathways such as the phosphatidylinositol-3-kinase (PI3K) and protein kinase B (PKB/AKT) [8,9]. One of the most frequently deleted genes in PCa, which negatively regulates PI3K-AKT signaling, is the tumor suppressor phosphatase and tensin homolog (PTEN) that is consistently associated with more aggressive forms and worse prognosis of PCa [10,11]. Loss of PTEN function has been well documented in PCa and PTEN mutations have been found in 40% metastatic PCa tumors [12,13]. Genetically Engineered Mouse Model (GEMM) *Pb-Cre4/Pten^f/f^* mice have been used since 2003, exhibiting pathological features similar with human prostate cancers, which includes the progression from intraepithelial neoplasia to invasive well- and poor-differentiated adenocarcinoma [14]. Moreover, this model has been used to produce new GEMM by combining mutations and to explore diet manipulation effects on prostate cancer progression [15,16].

Gene expression analysis is an important tool for understanding the behavior of tumors. Gene expression signatures have been successfully applied to define subclasses of different types of cancers with different biological behaviors and responses to therapies [17,18,19,20,21]. Several studies have revealed gene expression signatures of PCa tumors that correlate with poor prognosis in retrospective analyses [22,23,24]. Some of these molecular signatures help stratify patients with a Gleason score of 7, improve prognostic prediction, and provide appropriate management plans for patients after radical prostatectomy [23,24,25]. 

Comprehensive studies of histological, genomic, and transcriptome analyses and their relationship with PCa are necessary. Abida et. al. (2019) [26] presented an integrative analysis of genomic alterations with expression and histological evaluation of tumors from patients with mCRPC, representing the clinical spectrum of advanced disease, and with tissues collected before and after treatment with androgen signaling inhibitors [26]. However, most molecular signatures do not require validation before clinical use. In addition, some signatures include too many genes, which are expensive and hard to use in the clinic [23]. In addition, the list of genes generated in these signatures generally does not overlap between studies, and no gene sets have been validated for clinical use [27,28,29]. The search for molecular gene signatures is based on the assumption that a clear distinction between tumors that will relapse and those that will not is possible using gene expression profiles [29]. Therefore, more studies are needed to identify and validate prognostic markers.

Therapeutic and diagnostic options for PCa are limited, and progress in drug development is delayed because most cancers are highly complex at different levels, including cellular, genomic, and metabolic. The current challenge in PCa diagnosis is the lack of alternative screening to replace the existing PCa biomarker, prostate-specific antigen (PSA). Although PSA is widely used, it cannot distinguish between indolent and aggressive PCa [30,31,32]. Therefore, exploring new types of biomarkers beyond the conventional AR and PI3K pathways and/or altered genes, such as *PTEN*, *P53,* and *RB1,* are highly important in prostate cancer research. 

The tumor microenvironment plays an important role in the initiation and progression of tumors [33,34,35]. Transcriptome analysis revealed that stromal regions adjacent to the tumor express genes that allow for re-stratification of the tumor microenvironment [36]. Secreted and membrane proteins play an important role in cancer metastasis by stimulating cancer cell migration and invasion, consequently increasing cancer metastasis [35,36,37,38]. Therefore, investigating potential targets for diagnosis and prognosis that are available in PCa tumor stroma provides an opportunity to reframe and help treat this disease. 

In this study, we used available data to perform an integrative analysis of the PCa secretome and tumor membrane proteome. Our design consisted of identifying potential biomarker targets at different stages of PCa progression, demonstrated here by the early stages of mouse Prostatic Intraepithelial Neoplasia (mPIN), Middle-stage tumor (MT), and Advanced-stage tumor (AT), focusing on the tumor microenvironment of PCa. From the list of targets (genes and proteins) available in the Human Protein Atlas (HPA) secretome, we investigated a commonly deregulated gene network in the transcriptome of *Pb-Cre4/Pten^f/f^* mice. Enrichment analysis, protein–protein interaction (PPI) network, and *in silico* tools allowed us to identify nine membranes and secreted proteins that were either downregulated or upregulated in PCa. We also compared the transcriptomic profiles of prostate adenocarcinoma (PRAD) and normal tissue samples using The Cancer Genome Atlas (TCGA) and Genotype-Tissue Expression (GTEx) data, which revealed that four genes that encode secreted proteins were downregulated in PRAD. Finally, the gene expression patterns and prognosis of patients with PCa were analyzed by comparing four published datasets with disease outcomes (decreased relapse-free survival, overall survival, and probability of freedom from biochemical recurrence), with subsequent validation of HPA protein expression in human PCa and normal prostate samples.

## 2. Results

### 2.1. Identification of Gene Expression Profile in Prostate Cancer of Pb-Cre4/Pten^f/f^ Mice

We performed an integrative analysis of the prostate cancer secretome and membrane proteome data to identify clinically relevant diagnostic and prognostic biomarkers. According to the criteria used and from the list of genes included, our analysis identified upregulated and downregulated genes in the mPIN, MT, and AT distributed in all Anterior Prostate (AP), Dorsal Prostate (DP), Lateral Prostate (LP), and Ventral Prostate (VP) lobes. 

**Figure 1:**
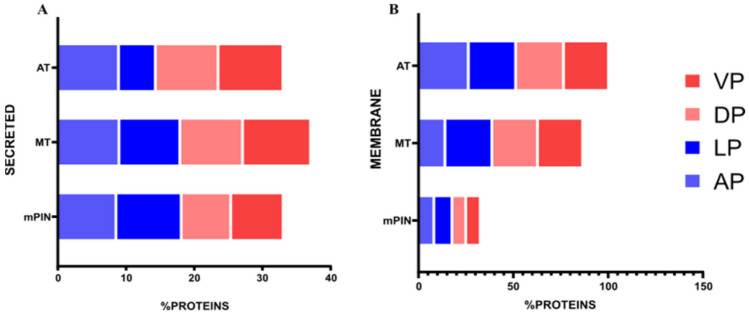
The proportion of membrane and secreted proteins downregulated and upregulated found in transcriptomics data of GEMM *Pb-Cre4/Pten^f/f^* PCa. (**A**) Bar plot showing the proportion of membrane protein in GEMM *Pb-Cre4/Pten^f/f^* PCa in VP, DP, LP, and AP; (**B**) Bar plot showing the proportion of secreted protein in GEMM *Pb-Cre4/Pten^f/f^* PCa in VP, DP, LP, and AP. VP = Ventral prostate; DP = Dorsal prostate; LP = Lateral prostate; AP = Anterior prostate. mPIN = mouse Prostatic Intraepithelial Neoplasia; MT = Middle-stage tumor; AT = Advanced-stage tumor.

Analysis of membrane protein targets showed upregulated genes: in the AP lobe (mPIN = 403 genes; MT = 510 genes; AT = 536 genes), DP lobe (mPIN = 263 genes; MT = 319 genes; AT = 377 genes), LP lobe (mPIN = 261 genes; MT = 369 genes; AT = 421 genes), and VP lobe (mPIN = 127 genes; MT = 316 genes; AT = 303 genes). The downregulated genes for membrane proteins were as follows: in the AP lobe (mPIN = 184 genes; MT = 237 genes; AT = 192 genes), DP lobe (mPIN = 146 genes; MT = 204 genes; AT = 347 genes), LP lobe (mPIN = 245 genes; MT = 264 genes; AT = 308 genes), and VP lobe (mPIN = 88 genes; MT = 207 genes; AT = 181 genes) (Figure 1A).

Analyses of the secreted protein targets were performed for each prostate lobe, and we found upregulated and downregulated genes in the three stages, mPIN, MT, and AT, of PCa progression. The number of upregulated genes was as follows: in the AP lobe (mPIN = 120 genes; MT = 155 genes; AT = 196 genes), DP lobe (mPIN = 49 genes; MT = 103 genes; AT = 163 genes), LP lobe (mPIN = 88 genes; MT = 141 genes; AT = 158 genes), and VP lobe (mPIN = 55 genes; MT = 128 genes; AT = 122 genes). The downregulated genes for secreted proteins were as follows: AP lobe (mPIN = 86 genes; MT = 100 genes; AT = 66 genes), DP lobe (mPIN = 69 genes; MT = 97 genes; AT = 101 genes), LP lobe (mPIN = 112 genes; MT = 87 genes; AT = 99 genes), and VP lobe (mPIN = 49 genes; MT = 69 genes; AT = 72 genes) (Figure 1B).

**Figure 2:**
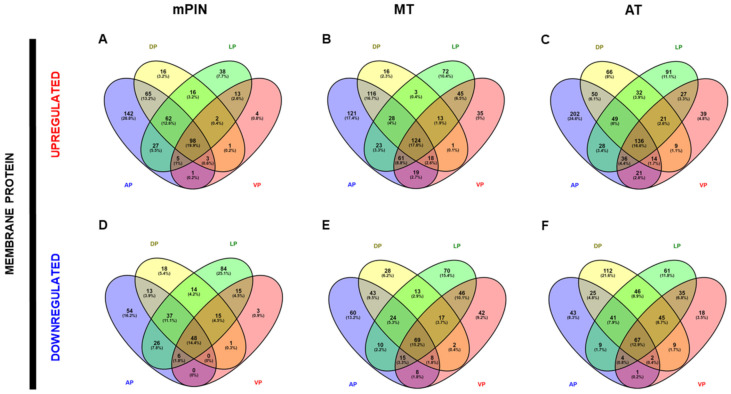
Venn’s diagrams depicting common genes of membrane proteins from Anterior Prostate (AP), Dorsal Prostate (DP), Lateral Prostate (LP), and Ventral Prostate (VP) PCa in *Pten* knockout mice. Venn’s diagrams of exclusive upregulated genes in mPIN (**A**), MT (**B**), and AT (**C**), and downregulated genes in mPIN (**D**), MT (**E**), and HT (**F**). mPIN = mouse Prostatic Intraepithelial Neoplasia. MT = Middle-stage tumor. AT = Advanced-stage tumor.

After identifying the list of upregulated and downregulated genes in each prostate lobe, we checked for shared genes present in the AP, DP, LP, and VP lobes. We identified a list of genes common to the four prostate lobes. The number of upregulated common genes for membrane proteins was as follows: in mPIN = 98 genes, MT = 124 genes, and AT = 136 genes (Figure 2A–C). The number of downregulated common genes for membrane proteins present in the four lobes was as follows: in mPIN = 48 genes, MT = 69 genes, and AT = 67 genes (Figure 2D–F). 

**Figure 3:**
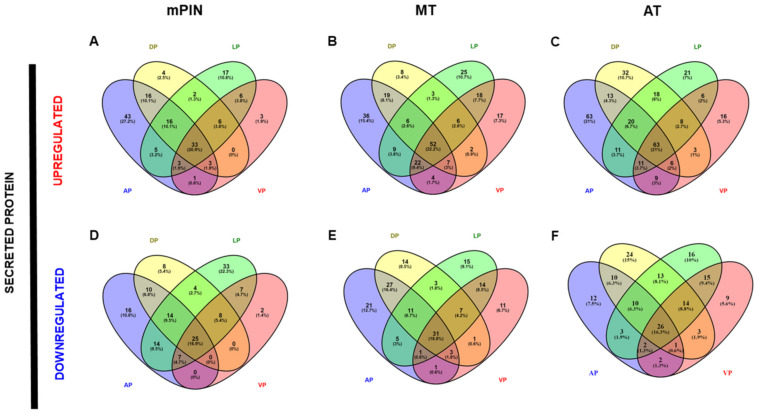
Venn’s diagrams depicting common genes of secreted proteins from Anterior Prostate (AP), Dorsal Prostate (DP), Lateral Prostate (LP), and Ventral Prostate (VP) PCa in *Pten* knockout mice. Venn’s diagrams of exclusive Upregulated genes in mPIN (**A**), MT (**B**), and AT (**C**), and downregulated genes in mPIN (**D**), MT (**E**), and HT (**F**). mPIN = mouse Prostatic Intraepithelial Neoplasia. MT = Middle-stage tumor. AT = Advanced-stage tumor.

A list of secreted protein genes common to all four prostate lobes was also performed. The number of genes commonly upregulated for secreted proteins was as follows: in mPIN = 33 genes, MT = 52 genes, and AT = 63 genes (Figure 3A–C). The number of downregulated common genes for secreted proteins present in the four lobes was as follows: in mPIN = 25 genes, MT = 31 genes, and AT = 26 genes (Figure 3D–F). 

Our list of downregulated and upregulated PCa genes in mPIN, MT, and AT was analyzed using the EnrichR platform to identify enriched ontological terms. The most significant categories were tyrosine kinase activity, an integral component of the plasma membrane for upregulated genes (Table 1 and Table 2). The most enriched terms for downregulated genes were inorganic cation transmembrane transport, potassium channel activity, and ion transmembrane transport (Table 3 and Table 4). This analysis also showed their involvement in biological processes, such as glycolysis, carbohydrate biosynthetic processes, glycosaminoglycan metabolic processes, and extracellular organization.

### 2.2. Protein–Protein Interaction (PPI) Network of Membrane and Secreted Proteins Enriched in Prostate Cancer

**Figure 4:**
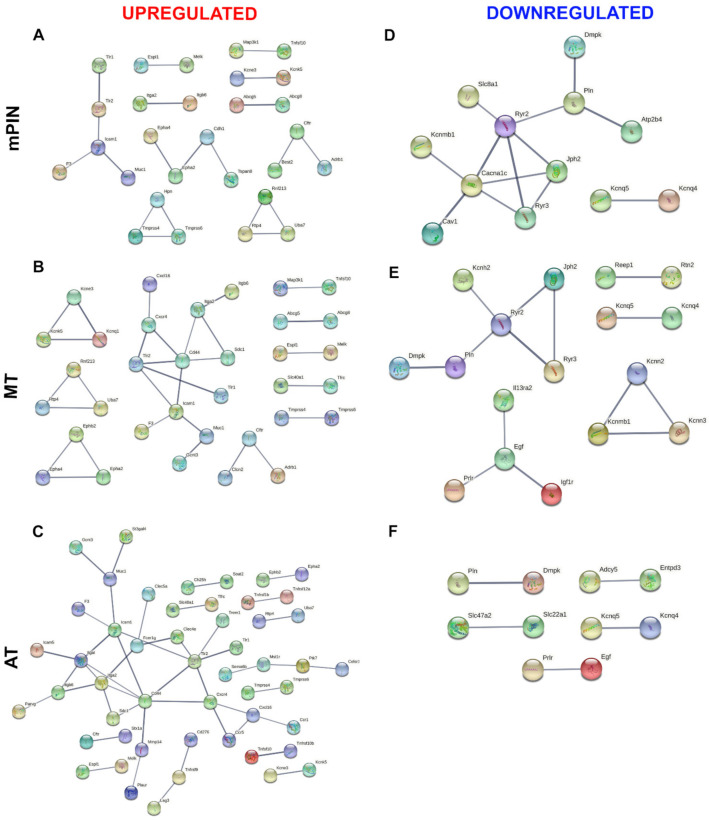
The PPI network involves common genes analysis of a cluster of membrane proteins of GEMM *Pb-Cre4/Pten^f/f^* PCa. PPI of commonly upregulated genes in mPIN (**A**), MT (**B**), and AT (**C**), and downregulated genes in mPIN (**D**), MT (**E**), and AT (**F**). Analysis using STRING [39] illustrates potential interactions. Proteins in the interaction network are represented as nodes connected by lines whose thickness reflects a confidence index higher than 0.7. mPIN = mouse Prostatic Intraepithelial Neoplasia; MT = Middle-stage tumor; AT = Advanced-stage tumor.

**Figure 5:**
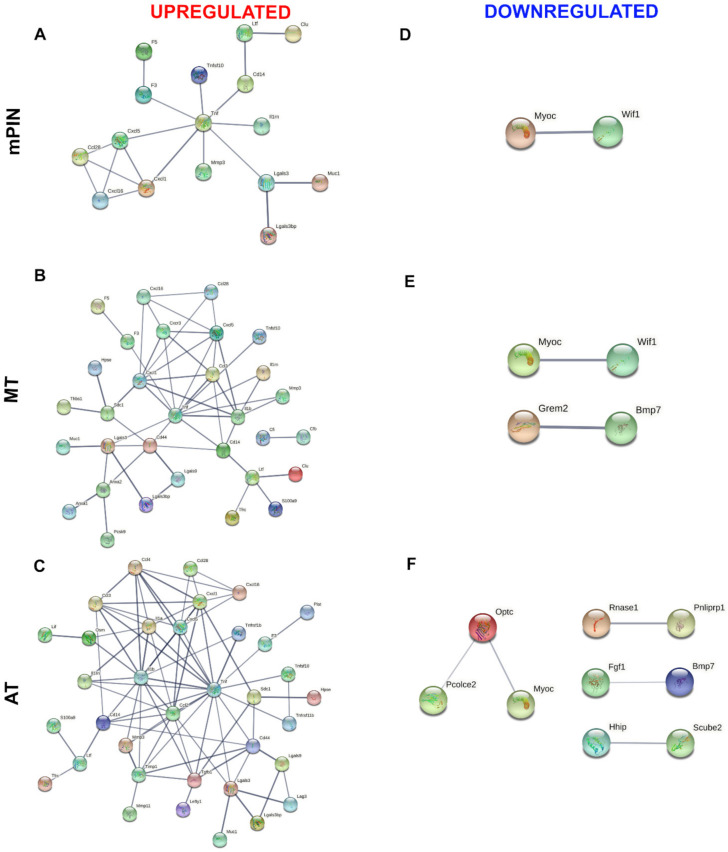
The PPI network involves common genes analysis of a cluster of secreted proteins of PCa in *Pten* knockout mice. PPI of commonly upregulated genes in mPIN (**A**), MT (**B**), and AT (**C**), and downregulated genes in mPIN (**D**), MT (**E**), and AT (**F**). Analysis using STRING [39] illustrates potential interactions. Proteins in the interaction network are represented as nodes connected by lines whose thickness reflects a confidence index higher than 0.7. mPIN = mouse Prostatic Intraepithelial Neoplasia; MT = Middle-stage tumor; AT = Advanced-stage tumor.

Venn diagrams showing the list of common genes identified in the four prostatic lobes of predicted membrane proteins are represented in Figure 2; upregulated and downregulated genes are shown in Figure 2A–C, and Figure 2D–F, respectively. The common genes identified in the predicted secreted proteins are represented in Figure 3 for upregulated (Figure 3A–C), and downregulated (Figure 3D–F) genes. Next, the common genes were used to generate a PPI network and GO using the STRING database (Supplementary Data). Data from this database revealed a complex interaction network among membrane proteins (Figure 4) and secreted proteins (Figure 5) with strong associations represented by thick lines of commonly upregulated genes in mPIN (Figure 4A and Figure 5A), MT (Figure 4B and Figure 5B), and AT (Figure 4C and Figure 5C); a complex interaction network with strong associations of commonly downregulated genes in mPIN (Figure 4D and Figure 5D), MT (Figure 4E and Figure 5E), and AT (Figure 4F and Figure 5F) is noted. The disconnected nodes in the network were hidden. 

GO analysis of upregulated membrane proteins revealed significant protein enrichment in the integral components of the membrane, cell surface, immune system processes, and cell surface receptor signaling pathways (Supplementary Materials Data; Figure S1). The GO terms of downregulated membrane proteins revealed significant protein enrichment in the sarcoplasmic reticulum membrane, transmembrane transporter activity, transmembrane transporter activity, and ion transport (Supplementary Materials; Figure S1).

GO analysis of upregulated secreted proteins revealed significant protein enrichment in the categories of immune system process, glycosaminoglycan metabolic process, and cell migration (Supplementary Materials; Figure S2). The GO terms of downregulated secreted proteins revealed significant protein enrichment in the extracellular region, glycosaminoglycan binding, and extracellular space (Supplementary Materials; Figure S2).

The PPI network for membrane proteins showed 28 (mPIN), 34 (MT), and 51 (AT) upregulated proteins and 12 (mPIN), 17 (MT), and 10 (AT) downregulated proteins (Figure 4). Whilst in the secreted proteins the PPI network showed 16 (mPIN), 31 (MT), and 34 (AT) upregulated proteins, while the PPI network showed 2 (mPIN), 4 (MT), and 9 (AT) downregulated proteins (Figure 5).

### 2.3. Differential Gene Expression of Transcripts Translated into The Membrane and Secreted Proteins in Prostate Cancer

After identifying all proteins present in the prostatic PPI network in mPIN, MT, and AT and among lobes, we selected the common upregulated and downregulated protein clusters in the three stages of PCa progression. The results identified 4 downregulated membrane proteins: Myotonin-protein kinase or myotonic dystrophy protein kinase (DMPK); Phospholamban (PLN); Potassium voltage-gated channel subfamily KQT member 5 (KCNQ5) and Potassium voltage-gated channel subfamily KQT member 4 (KCNQ4). We have also found 3 downregulated secreted proteins: Myocilin (MYOC); Wnt inhibitory factor 1 (WIF1); and Bone morphogenetic protein 7 (BMP7). The results identified Tissue Factor (F3) and Mucin-1 (MUC1) common upregulated proteins were present in secreted and membrane proteins. 

**Figure 6:**
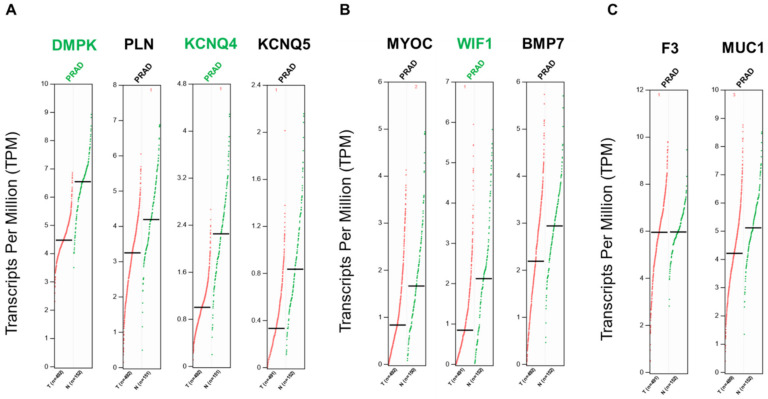
Differential gene expressions of transcripts translated into the membrane and secreted proteins in prostate cancer. (**A**)—Genes from downregulated membrane proteins. (**B**)—Genes from downregulated secreted proteins. (**C**)—Genes from upregulated proteins are commonly present in secreted and membrane proteins. Differential expression levels were calculated using the web based GEPIA tool [40]. GEPIA analysis revealed that genes of the proteins identified were negatively regulated in PRAD. Genes were considered positively or negatively regulated written in red and green, respectively, in PRAD (*n* = 489–492) relative to normal tissue (*n* = 150–152) when absolute values of fold-change were >1.0 and the *q*-value < 0.01 (ANOVA). Red dots: indicate prostate cancer tumor samples; green dots: indicate normal prostate samples.

The gene expression levels of the targets identified in our final list were analyzed using the online Gene Expression Profiling Interactive Analysis (GEPIA) tool (Tang et al., 2017). This tool allows the comparison of transcriptome profiles from TCGA and GTEx using uniformly processed and unified RNA sequencing data from the Toil Pipeline. The expression profiles of genes encoding secreted and membrane proteins were analyzed using the GEPIA tool to identify prognostic biomarkers of PRAD. The analysis showed that three genes (*DMPK*, *KCNQ4*, and *WIF1*) were significantly downregulated in PRAD (Log_2_ fold change cutoff = 1 and *q*-value cutoff = 0.01) when compared to normal tissues (Figure 6).

### 2.4. Survival Analysis and Risk Assessment

**Figure 7:**
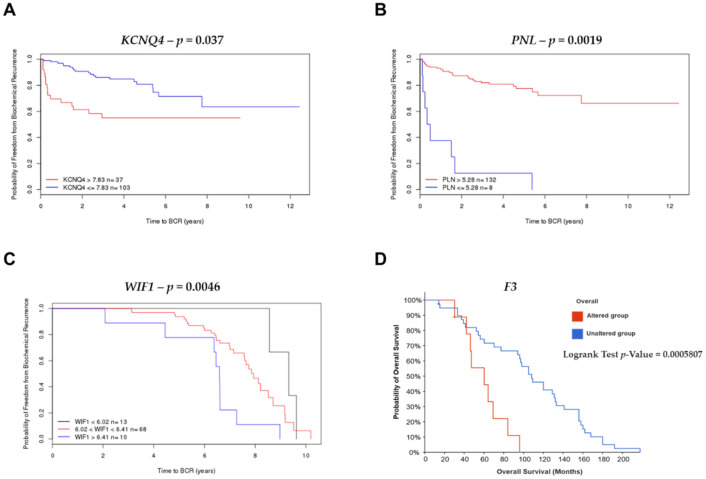
Kaplan–Meier curves displaying the probability of freedom from BRC and overall survival of PCa with (red) or without (blue) Differentially Expressed Genes (DEGs) overexpression. Analyzed by the Cambridge Carcinoma of the Prostate App (CamcAPP) (https://bioinformatics.cruk.cam.ac.uk/apps/camcAPP/) [41] (accessed on 21 December 2021) and the cBioPortal for Cancer Genomics database (https://www.cbioportal.org/) (accessed on 21 December 2021) [42,43] from an integrative study. (**A**)—Kaplan–Meier curve with the probability of freedom from biochemical recurrence of PCa with (red) or without (blue) *KCNQ4* overexpression with cut-off = 7.83 from the MSKCC study [8]; the difference was statistically significant, *p* = 0.037. (**B**)—Kaplan–Meier curve with the probability of freedom from biochemical recurrence of PCa with (red) or without (blue) *PLN* overexpression with cut-off = 5.28 from the MSKCC study [8]; the difference was statistically significant, *p* = 0.0019. (**C**)—Kaplan–Meier curve with the probability of freedom from biochemical recurrence of PCa with *WIF1* expression with cut-off <6.02 (black line), WIF1 gene expression with cut-off <6.41 (red line), and patients with WIF1 gene expression with cut-off >6.41 (blue line) (*p* = 0.0046) from the Stockholm study [44]; the difference was statistically significant, *p* = 0.0046. (**D**)—Kaplan–Meier curve with the probability of overall survival of PCa patients with (red) or without (blue) *F3* alteration from the metastatic prostate adenocarcinoma (MCTP, Nature 2012) study [45]; the difference was statistically significant, *p* = 0.0005807.

The gene (*KCNQ4*, *PNL*, *F3,* and *WIF1*) expression patterns and prognosis of patients with PCa were analyzed by comparing four published datasets (MSKCC, Cambridge, Stockholm, and MCTP). In these analyses, overexpression of the *KCNQ4* gene expression with cut-off >7.83 (red line) and £7.83 (blue line) was associated with a reduced time of biochemical recurrence (*p =* 0.037) in the MSKCC dataset (Figure 7A). In the MSKCC and Stockholm datasets *PLN* (*p* = 0.019) expression with cut-off >5.28 (red line) and £5.28 (blue line), and *WIF1* (*p =* 0.0046) overexpression with cut-off <6.41 (red line), and patients with WIF1 gene expression with cut-off >6.41 (blue line) were associated with a good prognosis for biochemical recurrence (Figure 7B,C). In metastatic PCa, the expression of *F3* showed the worst probability of overall survival (*p* = 0.0005807) in the metastatic prostate adenocarcinoma (MCTP, Nature 2012) study (Figure 7D). The other genes, *DMPK*, *KCNQ5*, *MYOC*, *BPM7*, and *MUC1* did not show statistical differences in the analysis of Kaplan–Meier curves for the probability of freedom from biochemical recurrence (BRC) and overall survival to PCa. A histogram of expression level of *KCNQ4*, *PLN* and *WIF1* were generated with a line to indicate the recursive partitioning (RP) cut-off (Supplementary Materials).

### 2.5. In Silico Validation of Protein Expression in Membrane and Secreted Proteins in Human Prostate Cancer

**Figure 8:**
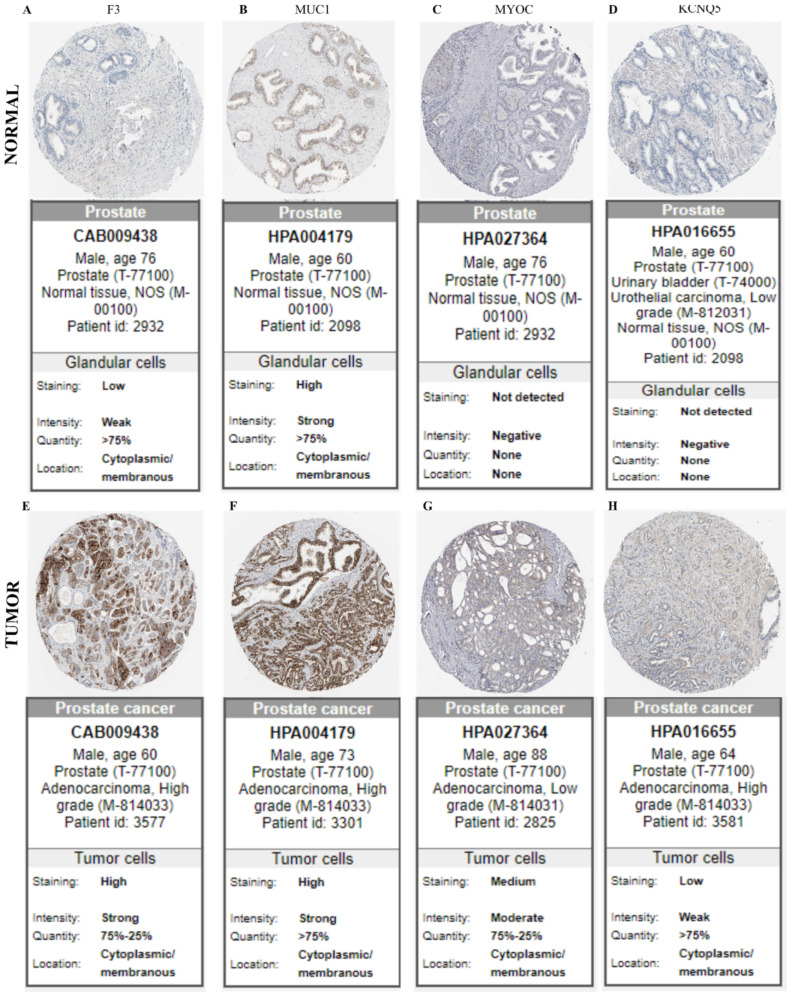
The validation of target genes in normal and tumor tissues. Immunostaining of normal and prostate tumor samples, respectively, for targets in our samples are: F3 (**A**,**E**); MUC1 (**B**,**F**); MYOC (**C**,**G**); and KCNQ5 (**D**,**H**), using immunohistochemical data available in the Human Protein Atlas database (https://proteinatlas.org/ accessed on 21 December 2021).

We analyzed the protein expression in human PCa and normal prostate samples from two upregulated (F3 and MUC1) and downregulated (MYOC and KCNQ5) targets found in the membrane and secreted protein list. The expression of F3 and MUC1 proteins using the HPA database showed increased (high or medium) immunostaining intensity in PCa tumor tissues; however, the expression remained low or undetectable in normal prostate tissues (Figure 8). Other dysregulated proteins identified in the HPA analyses were KCNQ4, DMPK, and PLN. At the time of this study, there was no immunohistochemistry tissue data available in the HPA database on PCa samples for BMP7 and WIF1 proteins. 

### 2.6. In Silico Prediction of miRNAs-mRNA Regulatory Modules in Prostate Cancer

**Figure 9:**
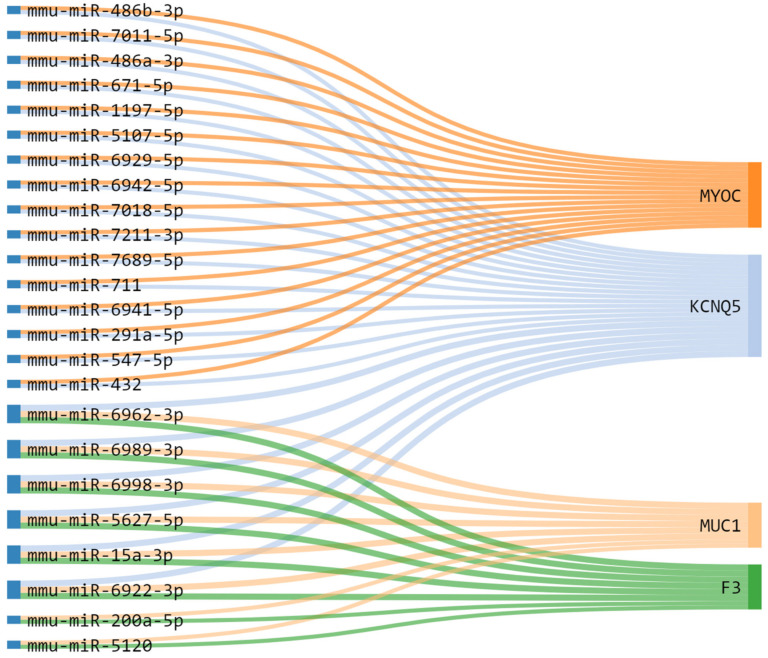
Dysregulated targets genes and potential association with micro-RNA (miRNA) in prostate tumor tissues. Alluvial diagram connecting the representative miRNA-mRNA targets, associated with genes clusters upregulated (*MUC1* and *F3*) and downregulated (*MYOC* and *KCNQ5*) in PRAD. Note the Highlight for mmu-miR-6962-3p, mmu-miR-6989-3p, mmu-miR-6998-3p, mmu-miR-5627-5p, mmu-miR-15a-3p, and mmu-miR-6922-3p regulating *MUC1*, *F3* and *KCNQ5*.

In order to obtain additional mechanistic information about the regulation of *MYOC*, *KCNQ5*, *MUC1*, and *F3*, we evaluated micro-RNAs (miRNAs) that can regulate the expression of these genes. After identifying the regulated miRNAs, using miRWalk 3.0, we selected only validated miRNAs interactions in at least three of the four DEGs. After constructing miRNA-mRNA interaction networks (Figure 9), we identified six miRNAs: mmu-miR-6962-3p, mmu-miR-6989-3p, mmu-miR-6998-3p, mmu-miR-5627-5p, mmu-miR-15a-3p, and mmu-miR-6922-3p that have a potential interaction that regulate three DEGs *KCNQ5*, *MUC1*, and *F3* in PCa (Figure 9). 

**Figure 10:**
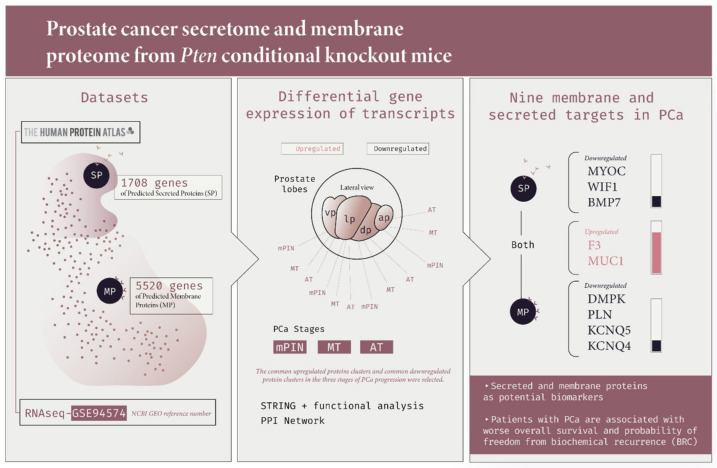
An overview of the main methodological strategies and results of prostate cancer secretome and membrane proteome from *Pten* conditional knockout mice and human prostate cancer. Here, we combined the selection of transcripts from the list of protein-coding genes of the secretome and membrane proteome dataset using The Human Protein Atlas Secretome and the *Pten* conditional knockout mice dataset (GSE 94574). After finding the upregulated and downregulated genes in the mPIN, MT and AT stages of PCa, we performed the GO enrichment analysis, PPI network using the STRING database, and the gene expression levels of the targets using the online GEPIA tool. Finally, we obtained nine (*DMPK*, *PLN*, *KCNQ5*, *KCNQ4*, *MYOC*, *WIF1*, *BMP7*, *F3*, and *MUC1*) deregulated PMPs and PSPs potential biomarker targets in PCa.

Figure 10 summarizes the main findings of the functional analysis and main results of our study.

## 3. Discussion

In this study, we first analyzed the PCa transcriptome from a *Pten* knockout mouse model for genes encoding membrane proteins and secreted proteins in PCa. We identified important DEGs in the PCa extracellular matrix (ECM) pertaining to hitherto unexplored pathways. From an integrative analysis of the secretome and membrane proteome, we identified 9 altered targets in PCa progression stages. The gene expression profile of these markers was altered in human PCa patients with worse overall survival and a worse probability of biochemical recurrence. Our strategy was to identify potential prognostic biomarker targets at different stages of PCa progression, focusing on the tumor microenvironment of PCa.

Tumors in patients with PCa present large histological, genetic, and molecular heterogeneity. A patient may harbor more than one genomic and phenotypically distinct prostate cancer; that is, these tumors appear independently and follow separate evolutionary trajectories. These clonally independent tumors exhibit biological differences and contribute differently to disease progression and clinical outcomes [46,47,48]. Currently, data on PCa proteomics and transcriptomes, using different GEMM and human patient samples, have been explored and integrated to identify potential targets against this disease.

The tumor microenvironment is a dynamic network of cells and structures, including tumor cells. The surrounding stroma is comprised of cancer-associated fibroblasts (CAFs), immune cells, mesenchymal stem cells (MSCs), ECM, cytokines, chemokines, and growth factors secreted by these cells [33,49]. It is already known that the tumor microenvironment plays an important role in the formation and progression of metastasis. CAFs deposit and degrade ECM components and thus remodel it during cancer progression, promoting immune cell infiltration and cancer cell proliferation, migration, and invasion [33,34]. CAFs can significantly promote proliferation and migration of prostate cancer cell lines [50,51]. Studies seeking to understand and identify precise biomarker signatures are necessary to identify effective targeted therapeutics to reduce the clinical and lethal diseases related to the role of inflammation in PCa progression [52,53]. It is important to note that studies that identified biomarkers for PCa, derived from markers of stromal infiltration or stromal transcriptomic and proteomic profiles, have not pointed to any of the markers that we found in our study [54,55,56,57]. Our targets are not directly related to the inflammatory profile but rather to another class of proteins related to the tumor microenvironment and ECM.

Of note, the findings of these targets were from the PCa transcriptome of knockout animals for *Pten* (*Pb-Cre4/Pten^f/f^* GEMM), in which they present important histopathological characteristics [58,59]. In addition to prostatic intraepithelial neoplastic (PIN) lesions, larger heterogeneous areas of fully invasive, both well- and poorly differentiated adenocarcinomas associated with reactive stroma are present in *Pb-Cre4/Pten^f/f^* GEMM. This model also presents a loss of the basal membrane structure and disorganization of the smooth muscle cells but shows rare metastasis. Additionally, infiltrated inflammatory cells are commonly identified in these tumors [58,59,60]. 

We believe that we found a set of proteins downregulated in PCa that are biologically important in (sub)types of human cancers. Myotonin-protein kinase or myotonic dystrophy protein kinase (DMPK) is a serine/threonine-protein kinase necessary for the maintenance of muscle structure and function [61]. DMPK is mainly expressed in smooth, skeletal, and cardiac muscles, and overexpression of DMPK mediated by p53 promotes contraction of the actomyosin cortex, which leads to the activation of caspases and concomitant cell death by apoptosis [61,62]. DMPK also phosphorylated phospholamban (PLN), another downregulated protein determined in our analyses. PLN is a small, and reversibly phosphorylated transmembrane protein found in the sarcoplasmic reticulum. Depending on its phosphorylation state, PLN binds to and regulates the activity of Ca^2+^ pumps [63]. These two proteins are downregulated during PCa progression. We believe that this was due to the loss of smooth muscle cells [58]. Our enrichment analysis also showed changes in the sarcoplasmic reticulum membrane and ion transport, which may be related to autophagy processes, calcium homeostasis, and endoplasmic reticulum stress, as previously reported [64,65].

We also identified potassium voltage-gated channel subfamily KQT member 5 (KCNQ5) and member 4 (KCNQ4), both of which are important in regulating neuronal excitability. Voltage-gated potassium channels are responsible for the repolarization phase of the membrane action potential and play crucial roles in the excitability of neurons and other cells (Li et al., 2021). Several studies have proposed the use of KCNQ5 gene for the early clinical detection of colorectal precancerous lesions and cancer [66,67,68]. Downregulated expression of KCNQ5 has also been observed in other diseases [69,70]. These proteins play an important role in potassium homeostasis and are related to the enriched terms of potassium channel activity and potassium ion transport at different levels of PCa progression presented in our results. In our analysis, patients with altered *KCNQ4* and *PLN* genes showed the shortest time for biochemical recurrence. The downregulation of these genes may be related to PCa progression.

In addition to membrane proteins, Myocilin (MYOC) was identified in our study. MYOC is a secreted glycoprotein that regulates the activation of different signaling pathways in adjacent cells to control different processes, including cell adhesion, cell-matrix adhesion, cytoskeleton organization, and cell migration [71]. Mutations in the *MYOC* gene are an important cause of glaucoma with dominant inheritance (Liuska [72,73]. However, other types of cancer, such as thymoma, exhibit MYOC downregulation, thereby corroborating our results [74]. 

Wnt inhibitory factor 1 (WIF1) is a secreted protein that binds to WNT proteins and inhibits their activities. WNT signaling mainly controls cell proliferation, differentiation, and maintenance of stem cells (β-catenin-dependent pathway), cell polarity, and migration (β-catenin-independent signaling). The WNT/Ca^2+^ signalling pathway is also associated with the release of Ca^2+^ from intracellular stores [75,76]. A large body of evidence has shown that activation of the WNT signaling pathway contributes to the proliferation and transformation of malignant cells with metastatic activity [77,78]. The WNT protein is regulated by a variety of secreted extracellular proteins that interfere with the formation of the WNT-receptor complexes. Extracellular inhibition of the WNT signaling pathway, WIF1, plays an important role in controlling cell proliferation and acts as a tumor suppressor [79]. Owing to its biological function, interest in using WIF1 as a biomarker for the early detection, diagnosis, and prognosis of cancer has increased in recent years [80,81,82,83]. As shown here, WIF1 was associated with favorable overall survival in PCa, corroborating other studies [84].

Bone morphogenetic protein 7 (BMP7) (https://www.uniprot.org/uniprot/P18075 (accessed on 21 December 2021)) is a growth factor of the TGF-β superfamily that plays important role in various biological processes, including proliferation, differentiation, and apoptosis in many different cell types [85,86]. Bone morphogenetic proteins can act as either tumor suppressors or oncogenes, depending on the cellular context and tumor type [87,88]. Studies have suggested that BPM7 inhibition may represent a target for overcoming resistance to cancer immunotherapies [85], and the use of BPM7 overexpression is a strong predictor of the risk of tumor recurrence in gastric cancer [87].

The upregulated gene in the common membrane and secreted proteins, tissue factor (F3) (https://www.uniprot.org/uniprot/P13726 (accessed on 21 December 2021)), is a transmembrane glycoprotein and primary initiator of the extrinsic blood coagulation cascade and ensures rapid hemostasis in case of organ damage [89]. F3 has been associated with strong tumor growth enhancement and poor prognosis in cancer [90]. In our analyses, we found that PCa patients with altered F3 gene expression had reduced survival rate. F3 expression is increased in tumors and is associated with tumor progression, particularly in pancreatic [91,92], cervical [93], breast [94], and prostate cancers [95]. 

The transmembrane glycoprotein Mucin-1 (MUC1) is highly glycosylated and is normally expressed in glandular and luminal epithelial cells. MUC1 provides protection and creates a physical barrier to negatively charged sugars, limiting accessibility, and preventing pathogenic colonization [96,97]. MUC1 is overexpressed and has been identified as a potential target for diagnosis, prognosis, and therapy in most human cancers and plays an important role in tumor progression [96,97,98,99,100]. Recently, we reported a family of deregulated mucins, including MUC1, in PCa progression, where it was shown that mucin cells (mucinous metaplasia) are in AR-negative areas of proliferation, and that mucin-associated genes have a worse prognosis in PCa and have significant prognostic value for PCa patients [58].

miRNA controls gene expression by targeting mRNA based on sequence complementarity and can serve as oncomiR or tumor suppressor miRs by targeting mRNA that encode oncoproteins or tumor suppressor proteins [101,102]. Using miRNA-mRNA tumor expression data, we identified deregulated miRNA that was validated in the regulatory networks of the four target genes. Some miRNAs found in our analysis that regulate *MYOC*, *KCNQ5*, *MUC1*, and *F3* mRNAs has been also described in other cancers, such as colorectal cancer cells [103] and identified as biomarkers in lung cancer [104], osteosarcoma [105], ovarian cancer [106] and penile cancer [107].

The miR-15a-3p miRNA has been associated with the three DEGs (*KCNQ5*, *MUC1*, and *F3*) in PCa in our analysis. The miR-15a-3p has been shown to suppress proliferation and migration inhibiting the expression of *BCL2* and *MCL1* in epithelial cells [108] and restrains the growth and metastasis of ovarian cancer cells by regulating *Twist1* [106]. Evidence has shown that miR-15a-3p overexpression also suppressed cell proliferation via down-regulating Wnt/β-catenin signaling in PCa cells [109]. Moreover, the mir-671-5p was previously described to function as a tumor suppressor, inhibiting tumor proliferation by blocking cell cycle in osteosarcoma and negatively regulates SMAD3 to inhibit migration and invasion of osteosarcoma cells [110,111]. The mir-671-5p interacts with *MYOC* and *KCNQ5* and may be down-regulating the expression of these genes in PCa.

Here, our strategy consisted of selecting upregulated and downregulated membrane and secreted proteins identified in PCa transcriptome analysis, which stratifies a group of unexplored proteins with high prognostic value and a potential target for therapy in a subgroup of patients. Although we tried to avoid bias in our study, certain limitations still need to be considered. Experimental in vivo and in vitro analysis should be performed to confirm our findings. Investigate the role and function of these miRNAs in PCa and their regulation of these genes are required. The experimental validation of the membrane and secreted proteins identified in this study could help correlate the results obtained herein with another group of patients’ prognoses, diagnosis, and/or overall survival. Despite the above limitations, we have demonstrated a well-characterization of secretome and membrane proteome and have spotted new dysregulated target candidates in PCa. 

## 4. Materials and Methods

### 4.1. Analysis of RNA-Seq Data of the Genetically Engineered Mouse Model (GEMM) for PCa: The Pten Conditional Knockout

We used RNA-seq data derived from the analysis of samples from the four prostatic lobes obtained from the GEMM *Pten^f/f^*, control, and *Pb-Cre4/Pten^f/f^* mice. We accessed RNA sequencing data derived from all prostate lobes using the NCBI Gene Expression Omnibus platform (GEO, https://www.ncbi.nlm.nih.gov/geo/ (accessed on 21 December 2021)), reference number GSE94574. Briefly, 72 samples were submitted for RNA-seq analysis, including 20 prostate samples from wild-type (WT), 16 mouse prostatic intraepithelial neoplasia (mPIN), 20 well-differentiated tumors (middle-stage tumor, MT), and 16 poorly differentiated tumors (advanced-stage tumor, AT). A minimum of four samples for each prostatic lobe and pathological condition for RNA-seq analysis were used. A detailed description of the histopathological aspects of each prostatic lobe and tumor stage of the mouse model has been previously described [59,112]. First, we explored genes that were differentially expressed in each lobe and at different stages of tumor progression (mPIN, MT and AT); we used Log_2_FC ≥ |+1| ≤ |−1| and adjusted the *p*-value < 0.05. The transcriptome used in this study was generated from animals provided by Dr. David Neal with experiments approved by the CRUK Institute Ethics Committee of the Cambridge University, UK, under design license 80/2435, and by the Ethics Committee on Animal Experimentation of the Institute of Biosciences of Botucatu (IBB)—UNESP, Brazil (Protocol CEUA 613/2014, 1145/2019 and 4921200721).

### 4.2. Integration of Secretome and Membrane Proteome Analyses to Identify Prostate Cancer Biomarkers

The DEGs from RNA-seq were used to predict membrane and secretome proteins using a known list of 5520 genes of Predicted Membrane Proteins and 1708 genes of Predicted Secreted Proteins available in The Human Protein Atlas Secretome (https://www.proteinatlas.org/humanproteome/tissue/secretome) [113,114] (accessed on 21 December 2021). The Predicted Membrane Proteins are a selection of seven prediction algorithms used to create a majority decision-based method (MDM) using the combined results from the chosen tools to estimate the human membrane proteome [115]. The human secretome was predicted by a whole-proteome scan using three methods for signal peptide prediction: *SignalP4.0*, *Phobius*, and *SPOCTOPUS*, which have all been shown to produce reliable prediction results in comparative analysis and selected the genes that were altered in at least three prostatic lobes in mPIN, MT, and/or AT.

### 4.3. Protein–Protein Interaction Network and Functional Enrichment Analysis

We used EnrichR software from Ma’ayan Lab (https://maayanlab.cloud/Enrichr/) (accessed on 21 December 2021) to determine the enrichment of ontological terms and molecular pathways related to DEGs [116,117]. The cutoff criteria used for both analyses were adjusted to a *p*-value of ≤ 0.05. Gene ontology (GO) enrichment analysis of secreted and membrane proteins was grouped into a single list. The ontological terms downregulated and upregulated in the biological process, molecular function, and cellular component categories with the lowest adjusted *p*-values were selected.

We used the STRING database (https://string-db.org/) [39] to identify the protein–protein interaction (PPI) network by individually analyzing the upregulated and downregulated genes. The minimum interaction score required was 0.700 (high confidence), and the nodes disconnected from the network were hidden to simplify the display. The PPI enrichment *p*-value indicated the statistical significance provided by STRING (accessed on 21 December 2021). 

The ShinyGO application (version 0.741) (http://bioinformatics.sdstate.edu/go/) [118] (accessed on 21 December 2021) was used to explore the enrichment of ontological terms in GO (http://geneontology.org/) (accessed on 21 December 2021) categories for the biological process of proteins from PPI. The cut-off criterion used for both analyses was a false discovery rate (FDR) *p*-value < 0.05.

### 4.4. Gene Expression Profile in Prostate Cancer

Differential expression levels were calculated using a web-based gene expression profiling analysis (GEPIA) tool [40]. GEPIA analysis revealed that genes encoding secreted proteins are regulated in PRAD (http://gepia.cancer-pku.cn/detail.php) (accessed on 21 December 2021). DEGs between tumor and normal samples were determined by one-way analysis of variance (ANOVA), applying the log_2_ fold-change > 1 and *q*-value < 0.01. Genes were considered positively or negatively regulated and indicated in red and green, respectively, in PRAD (*n =* 489–492) relative to normal tissue (*n* = 150–152).

### 4.5. Survival Analysis and Risk Assessment

After identifying the secretome and membrane proteome targets of knockout mice, we performed analyses using data from publicly available databases. We investigated gene expression using the Cambridge Carcinoma of the Prostate App (CamcAPP) database and developed the CamcAPP (https://bioinformatics.cruk.cam.ac.uk/apps/camcAPP/) [41] (accessed on 21 December 2021) and the cBioPortal for Cancer Genomics database (https://www.cbioportal.org/) [42,43] (accessed on 21 December 2021), to determine the association of gene alterations with patient clinical data, such as tumor risk development, prognosis, and survival rates. Survival curves were constructed using the Kaplan–Meier method. The expression of genes was associated with disease outcomes (decreased relapse-free survival and an increased expression level of genes in advanced prostate cancer) in several published PCa datasets, namely Memorial Sloan-Kettering Cancer Center (MSKCC) [8] and Cambridge and Stockholm integrative studies [44] performed using CamcAPP [41]. The published PCa datasets used was Metastatic Prostate Adenocarcinoma (MCTP, Nature 2012) [45] performed using the cBioPortal for Cancer Genomics database [42,43]. The grouping of samples found by recursive partitioning (RP) was used to construct a Kaplan-Meier plot by CamcAPP.

### 4.6. In Silico Validation of Differentially Expressed Genes (DEGs)

After gene expression analysis, the deregulated genes identified in our analysis of GEMM *Pb-Cre4/Pten^f/f^* PCa were assessed using the HPA (https://www.proteinatlas.org/) database (accessed on 21 December 2021) [113,114] to identify the distribution and localization of proteins in normal and tumor prostate samples via immunohistochemistry.

### 4.7. Prediction of Commonly Dysregulated miRNAs-mRNA Targets

We used the miRWalk 3.0 tool (http://mirwalk.umm.uni-heidelberg.de/) [119] to perform the regulatory interaction between miRNA and mRNA (*MYOC*, *KCNQ5*, *MUC1*, and *F3*); the algorithm for target validation was used in other available databases of *Homo sapiens*. The miRNA was considered significant when involved with at least three of the four genes selected. An alluvial plot diagram was generated using the online tool SankeyMATIC (http://sankeymatic.com/) to demonstrate the interaction networks between the miRNA and mRNA.

### 4.8. Data Representation and Analysis

Venn diagrams were plotted using Venny 2.1 (https://bioinfogp.cnb.csic.es/tools/venny/) [120]. The STRING database was used (https://string-db.org/) [39] to construct a protein-protein interaction network. Differential expression levels were calculated using the web based GEPIA [40] (http://gepia.cancer-pku.cn/detail.php). The CamcAPP database (https://bioinformatics.cruk.cam.ac.uk/apps/camcAPP/) [41] and cBioPortal for Cancer Genomics database (https://www.cbioportal.org/) [42,43] were used for survival analysis. 

## Figures and Tables

**Table 1:** A gene ontology (GO) enrichment analysis of membrane proteins upregulated in mPIN, MT, and AT of prostate cancer.

**mPIN Upregulated Gene Ontology (GO)**				
**Pathway ID**	**Pathway Description**	**Gene Count**	**Adjusted *p*-Value**	**Log_10_**
GO:0005887	Integral component of plasma membrane	32	1.73 × 10^−11^	10.76299509
GO:0004713	Protein tyrosine kinase activity	5	0.012138352	1.915840284
GO:0016758	Hexosyltransferase activity	5	0.014297294	1.844746144
GO:0043190	ATP-binding cassette (ABC) transporter complex	2	0.019180885	1.717131357
GO:0008194	UDP-glycosyltransferase activity	4	0.024662147	1.607969117
GO:0009312	Oligosaccharide biosynthetic process	3	0.034162979	1.466444268
GO:0016051	Carbohydrate biosynthetic process	3	0.034162979	1.466444268
GO:0019375	Galactolipid biosynthetic process	2	0.034162979	1.466444268
GO:0006682	Galactosylceramide biosynthetic process	2	0.034162979	1.466444268
GO:0046476	Glycosylceramide biosynthetic process	2	0.0382352	1.417536631
GO:0006681	Galactosylceramide metabolic process	2	0.041917255	1.377607169
GO:0030165	PDZ domain binding	3	0.048747995	1.31204324
**MT Upregulated Gene Ontology (GO)**				
**Pathway ID**	**Pathway Description**	**Gene Count**	**Adjusted *p*-Value**	**Log_10_**
GO:0005887	Integral component of plasma membrane	43	9.77 × 10^−17^	16.01024386
GO:0016323	Basolateral plasma membrane	10	0.00000211	5.675736848
GO:0004713	Protein tyrosine kinase activity	7	0.001099549	2.9587853
GO:0019199	Transmembrane receptor protein kinase activity	5	0.002610742	2.5832361
GO:0004714	Transmembrane receptor protein tyrosine kinase activity	5	0.002610742	2.5832361
GO:0005254	Chloride channel activity	5	0.00268003	2.571860416
GO:0045121	Membrane raft	7	0.002867281	2.542529695
GO:0043005	Neuron projection	11	0.01394385	1.855617311
GO:0007167	Enzyme linked receptor protein signaling pathway	7	0.024380271	1.612961464
**AT Upregulated Gene Ontology (GO)**				
**Pathway ID**	**Pathway Description**	**Gene Count**	**Adjusted *p*-Value**	**Log_10_**
GO:0005887	Integral component of plasma membrane	46	1.74 × 10^−17^	16.75829697
GO:0030667	Secretory granule membrane	13	0.00000273	5.564593362
GO:0006954	Inflammatory response	11	0.000308	3.511189697
GO:0071345	Cellular response to cytokine stimulus	15	0.000421	3.375939369
GO:0002283	Neutrophil activation involved in immune response	13	0.00509802	2.292598474
GO:0004713	Protein tyrosine kinase activity	6	0.00518138	2.285554596
GO:0070372	Regulation of ERK1 and ERK2 cascade	9	0.005657676	2.247361946
GO:0032103	Positive regulation of response to external stimulus	7	0.006311353	2.199877562
GO:0043410	Positive regulation of MAPK cascade	9	0.008909311	2.050155885
GO:0030198	Extracellular matrix organization	9	0.012342256	1.908605459
GO:0005925	Focal adhesion	9	0.013649783	1.864874257
GO:0005789	Endoplasmic reticulum membrane	12	0.028337729	1.547634959

**Table 2:** A gene ontology (GO) enrichment analysis of secreted proteins upregulated in mPIN, MT, and AT of prostate cancer.

**mPIN Upregulated Gene Ontology (GO)**				
**Pathway ID**	**Pathway Description**	**Gene Count**	**Adjusted *p*-Value**	**Log_10_**
GO:0019221	Cytokine-mediated signaling pathway	10	0.0000154	4.813747101
GO:0062023	Collagen-containing extracellular matrix	7	0.0000354	4.451038902
GO:0016019	Peptidoglycan immune receptor activity	2	0.000428	3.368891105
GO:1902533	Positive regulation of intracellular signal transduction	7	0.001214858	2.915474599
GO:0042834	Peptidoglycan binding	2	0.002962979	2.528271463
GO:0008284	Positive regulation of cell population proliferation	6	0.003344125	2.475717449
GO:0001774	Microglial cell activation	2	0.008863214	2.052408777
GO:0030855	Epithelial cell differentiation	3	0.008882339	2.051472641
GO:0033628	Regulation of cell adhesion mediated by integrin	2	0.01496553	1.824907884
GO:0030198	Extracellular matrix organization	4	0.01496553	1.824907884
GO:0010811	Positive regulation of cell-substrate adhesion	2	0.037254018	1.428826881
GO:0060252	Positive regulation of glial cell proliferation	1	0.04201877	1.376556662
GO:0033631	Cell-cell adhesion mediated by integrin	1	0.04201877	1.376556662
GO:0071902	positive regulation of protein serine/threonine kinase activity	2	0.046363298	1.333825674
**MT Upregulated Gene Ontology (GO)**				
**Pathway ID**	**Pathway Description**	**Gene Count**	**Adjusted *p*-Value**	**Log_10_**
GO:0034774	Secretory granule lumen	14	4.44 × 10^−12^	11.35278878
GO:0006954	Inflammatory response	13	2.06 × 10^−11^	10.6853178
GO:0062023	Collagen-containing extracellular matrix	12	9.21 × 10^−9^	8.035890283
GO:1905517	Macrophage migration	3	0.000185	3.732564754
GO:0007160	Cell-matrix adhesion	3	0.011210456	1.950376724
GO:0030855	Epithelial cell differentiation	3	0.011480631	1.940034241
GO:0098632	Cell-cell adhesion mediator activity	2	0.025492739	1.5935835
GO:0030203	Glycosaminoglycan metabolic process	2	0.031920127	1.495935395
GO:0045545	Syndecan binding	1	0.045268101	1.344207727
GO:0048708	Astrocyte differentiation	1	0.048279814	1.316234416
GO:0008347	Glial cell migration	1	0.048279814	1.316234416
**AT Upregulated Gene Ontology (GO)**				
**Pathway ID**	**Pathway Description**	**Gene Count**	**Adjusted *p*-Value**	**Log_10_**
GO:0048018	Receptor ligand activity	19	3.70 × 10^−18^	17.43149267
GO:0019221	Cytokine-mediated signaling pathway	23	5.23 × 10^−16^	15.28185137
GO:0006954	Inflammatory response	15	1.64 × 10^−13^	12.78451214
GO:0062023	Collagen-containing extracellular matrix	13	1.23 × 10^−8^	7.90940544
GO:0008284	Positive regulation of cell population proliferation	13	2.40 × 10^−7^	6.620158329
GO:0070374	Positive regulation of ERK1 and ERK2 cascade	9	3.14 × 10^−7^	6.503756602
GO:0030198	Extracellular matrix organization	7	0.0000857	4.066941739
GO:0000165	MAPK cascade	7	0.0000906	4.043108741
GO:1905517	Macrophage migration	2	0.000194	3.712020847
GO:0043062	Extracellular structure organization	5	0.000676	3.170015558
GO:0042834	Peptidoglycan binding	1	0.006637813	2.177975013

**Table 3:** A gene ontology (GO) enrichment analysis of membrane proteins downregulated in mPIN, MT, and AT of prostate cancer.

**mPIN Downregulated Gene Ontology (GO) **				
**Pathway ID**	**Pathway Description**	**Gene Count**	**Adjusted *p*-Value**	**Log_10_**
GO:0098662	Inorganic cation transmembrane transport	11	2.10 × 10^−8^	7.677498224
GO:0005887	Integral component of plasma membrane	19	2.38 × 10^−8^	7.623546608
GO:0016529	Sarcoplasmic reticulum	6	3.99 × 10^−8^	7.399562462
GO:0042383	Sarcolemma	6	6.56 × 10^−8^	7.18327432
GO:0051480	Regulation of cytosolic calcium ion concentration	8	3.05 × 10^−7^	6.516291269
GO:0006874	Cellular calcium ion homeostasis	7	0.00000312	5.505384291
GO:0070588	Calcium ion transmembrane transport	5	0.0000635	4.197087214
GO:0005267	Potassium channel activity	4	0.002683985	2.571219909
GO:0005217	Intracellular ligand-gated ion channel activity	2	0.004871735	2.312316306
GO:0006939	Smooth muscle contraction	2	0.005857764	2.232268112
GO:0005790	Smooth endoplasmic reticulum	2	0.007141408	2.146216169
GO:0015081	Sodium ion transmembrane transporter activity	2	0.017072923	1.76769213
**MT Downregulated Gene Ontology (GO)**				
**Pathway ID**	**Pathway Description**	**Gene Count**	**Adjusted *p*-Value**	**Log_10_**
GO:0005267	Potassium channel activity	9	3.84 × 10^−9^	8.415844939
GO:0006813	Potassium ion transport	10	8.24 × 10^−9^	8.084282366
GO:0016529	Sarcoplasmic reticulum	7	9.40 × 10^−9^	8.027036253
GO:0005887	Integral component of plasma membrane	20	0.00000197	5.704434553
GO:0005789	Endoplasmic reticulum membrane	11	0.000418	3.37836809
GO:0015085	Calcium ion transmembrane transporter activity	3	0.010602036	1.974610737
GO:0005790	Smooth endoplasmic reticulum	2	0.018509381	1.732608106
GO:0005355	Glucose transmembrane transporter activity	2	0.019938461	1.700308368
**AT Downregulated Gene Ontology (GO)**				
**Pathway ID**	**Pathway Description**	**Gene Count**	**Adjusted *p*-Value**	**Log_10_**
GO:0005887	Integral component of plasma membrane	25	2.28 × 10^−10^	9.642075486
GO:0098662	Inorganic cation transmembrane transport	13	1.98 × 10^−9^	8.703210974
GO:0006813	Potassium ion transport	10	2.03 × 10^−9^	8.693400143
GO:0010232	Vascular transport	6	0.0000439	4.357272375
GO:0035725	Sodium ion transmembrane transport	6	0.0000439	4.357272375
GO:0005267	Potassium channel activity	6	0.0001	3.999508817
GO:0016529	Sarcoplasmic reticulum	4	0.000734	3.134317147
GO:0015079	Potassium ion transmembrane transporter activity	3	0.003470428	2.459616983

**Table 4:** A gene ontology (GO) enrichment analysis of secreted proteins downregulated in mPIN, MT and AT of prostate cancer.

**mPIN Downregulated Gene Ontology (GO)**				
**Pathway ID**	**Pathway Description**	**Gene Count**	**Adjusted *p*-Value**	**Log_10_**
GO:0062023	Collagen-containing extracellular matrix	6	0.000153	3.814225913
GO:0008237	Metallopeptidase activity	3	0.008113586	2.090787142
GO:0032027	Myosin light chain binding	1	0.049310437	1.307061149
GO:0008270	Zinc ion binding	3	0.049310437	1.307061149
**MT Downregulated Gene Ontology (GO)**				
**Pathway ID**	**Pathway Description**	**Gene Count**	**Adjusted *p*-Value**	**Log_10_**
GO:0062023	Collagen-containing extracellular matrix	7	0.0000450	4.34654643
GO:0005604	Basement membrane	2	0.037653103	1.424199233
GO:1903561	extracellular vesicle	2	0.037653103	1.424199233
**AT Downregulated Gene Ontology (GO)**				
**Pathway ID**	**Pathway Description**	**Gene Count**	**Adjusted *p*-Value**	**Log_10_**
GO:0062023	Collagen-containing extracellular matrix	6	0.000226	3.645111972
GO:0001823	Mesonephros development	2	0.028927408	1.538690472
GO:0004518	Nuclease activity	2	0.048024251	1.318539402
GO:0004540	Ribonuclease activity	2	0.048024251	1.318539402

## Data Availability

The results presented in this article are partly based on data generated by the Cambridge Carcinoma of the Prostate App (CamcAPP) (https://bioinformatics.cruk.cam.ac.uk/apps/camcAPP/) from the Cancer Research UK Cambridge Institute, cBioPortal for Cancer Genomics database (https://www.cbioportal.org/), and Human Protein Atlas (HPA) (https://www.proteinatlas.org/) database. The RNA sequencing data derived from all prostatic lobes of Pten knockout are available in the NCBI Gene Expression Omnibus platform (GEO, https://www.ncbi.nlm.nih.gov/geo/, accessed on 21 December 2021), reference number GSE94574. The datasets presented in this study can be found in online repositories on gene expression profiling interactive analysis (GEPIA) (http://gepia.cancer-pku.cn/index.html, accessed on 21 December 2021).

## Supplementary Materials

The supporting information can be downloaded at: https://www.mdpi.com/article/10.3390/ijms23169224/s1. Reference [121] are cited in the supplementary materials.
