# Supplementary material for: Prostate Cancer Secretome and Membrane Proteome from Pten Conditional Knockout Mice Identify Potential Biomarkers for Disease Progression
Source: Int J Mol Sci. 2022 Aug 17;23(16):9224. doi: 10.3390/ijms23169224 (PMC9409251; doi:10.3390/ijms23169224)
Supplement: Supplementary file 1 [file ijms-23-09224-s001.zip › ijms-1847392-Supplementary Files.pdf]

Supplementary Files

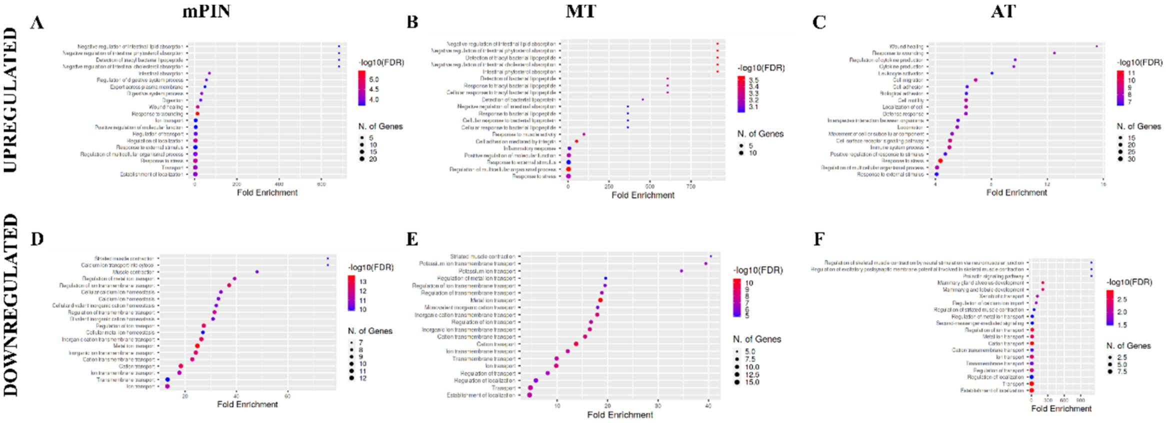

Figure S1: Functional enrichment analysis of a cluster of membrane proteins of PCa in Pten knockout mice. The selected top biological process from ORA analysis of membrane proteome from PPI in mPIN (A), MT (B), and AT (C), and downregulated genes in mPIN (D), MT (E), and AT (F).

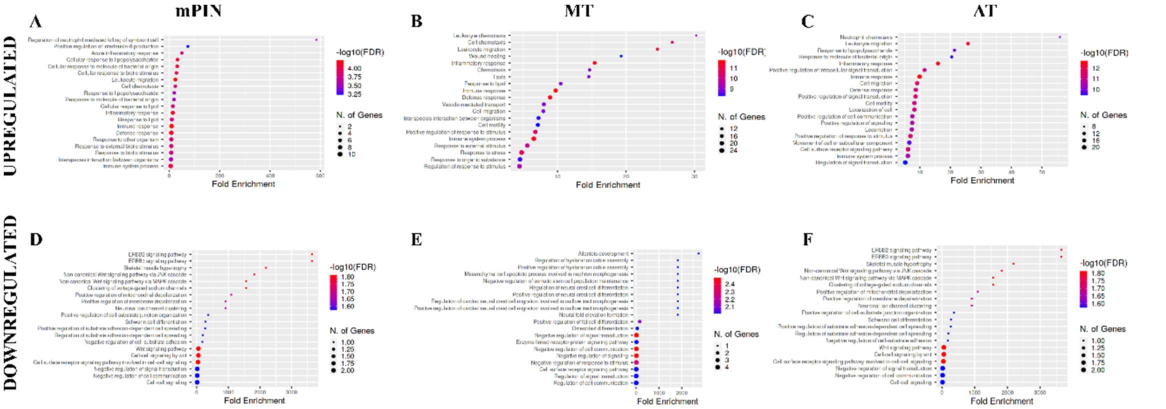

Figure S2: Functional enrichment analysis of a cluster of secreted proteins of PCa in *Pten* knockout mice. The selected top biological process from ORA analysis of membrane proteome from PPI in mPIN (A), MT (B), and AT (C), and downregulated genes in mPIN (D), MT (E), and HT (F).

Complementary results of the survival analyze and *p-value* and Recursive Partitioning (RP) cut-off

Recursive Partitioning analysis from The CamcAPP – Survival Analysis

A histogram of recursive partitioning (RP) analysis to *KCNQ4*, *PLN*, and *WIF1* genes has provided below. RP analysis is performed to determine if the samples can be split into groups based on the expression data from the chosen gene(s). An RP p-value < 0.05 indicates a significant split. A histogram of expression level of *KCNQ4*, *PLN*, and *WIF1* genes with a line indicate the RP cut-off [1]. The grouping of samples found by RP were used to construct a Kaplan-Meier plot (see in the main text, Fig. 7A, 7B, 7C).

	Gene	RP_p.value	RP_Cut.off
1	**KCNQ4**	0.037	7.83

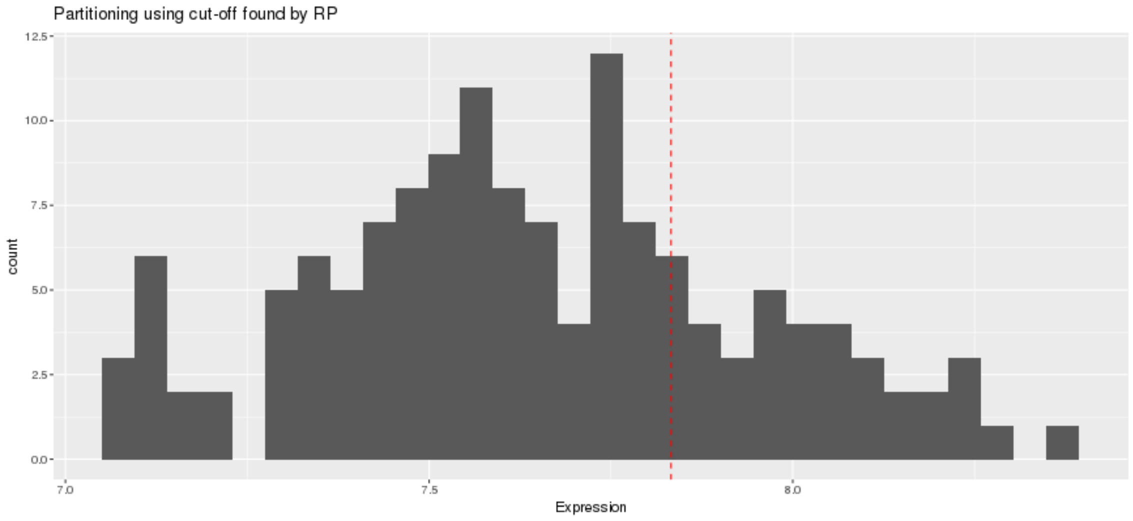

	Gene	RP_p.value	RP_Cut.off
2	**PLN**	0.0019	5.28

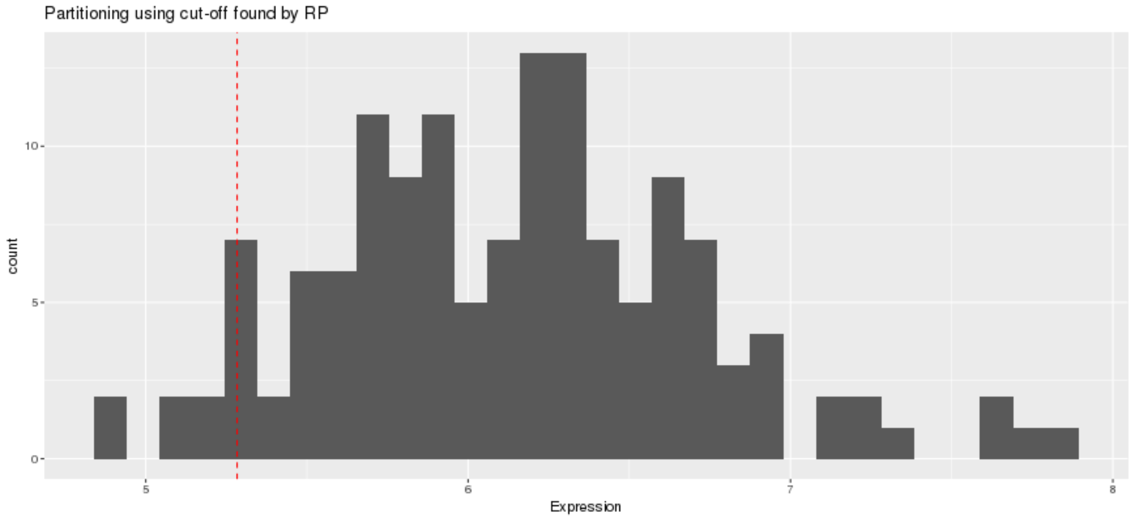

	Gene	RP_p.value	RP_Cut.off
3	WIF1	0.0046	6.02

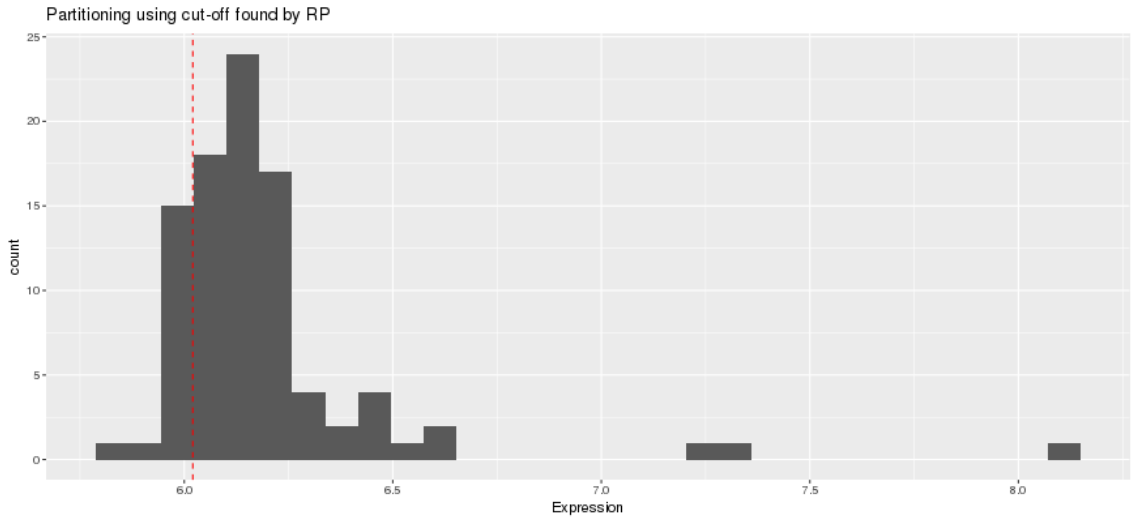

[1] Torsten Hothorn, Kurt Hornik and Achim Zeileis (2006). Unbiased Recursive Partitioning: A Conditional Inference Framework. Journal of Computational and Graphical Statistics, 15(3), 651--674.
